# Ebselen and Analogues: Pharmacological Properties and Synthetic Strategies for Their Preparation

**DOI:** 10.3390/molecules26144230

**Published:** 2021-07-12

**Authors:** Claudio Santi, Cecilia Scimmi, Luca Sancineto

**Affiliations:** Group of Catalysis and Green Organic Chemistry, Department of Pharmaceutical Sciences, University of Perugia Via del Liceo 1, 06122 Perugia, Italy; claudio.santi@unipg.it (C.S.); cecilia.scimmi@studenti.unipg.it (C.S.)

**Keywords:** ebselen, selenorganic compounds, antiviral, bipolar disorders, hearing loss, selenium chemistry

## Abstract

Ebselen is the leader of selenorganic compounds, and starting from its identification as mimetic of the key antioxidant enzyme glutathione peroxidase, several papers have appeared in literature claiming its biological activities. It was the subject of several clinical trials and it is currently in clinical evaluation for the treatment of COVID-19 patients. Given our interest in the synthesis and pharmacological evaluation of selenorganic derivatives with this review, we aimed to collect all the papers focused on the biological evaluation of ebselen and its close analogues, covering the timeline between 2016 and most of 2021. Our analysis evidences that, even if it lacks specificity when tested in vitro, being able to bind to every reactive cysteine, it proved to be always well tolerated in vivo, exerting no sign of toxicity whatever the administered doses. Besides, looking at the literature, we realized that no review article dealing with the synthetic approaches for the construction of the benzo[*d*][1,2]-selenazol-3(2*H*)-one scaffold is available; thus, a section of the present review article is completely devoted to this specific topic.

## 1. Introduction

**Figure 1:**
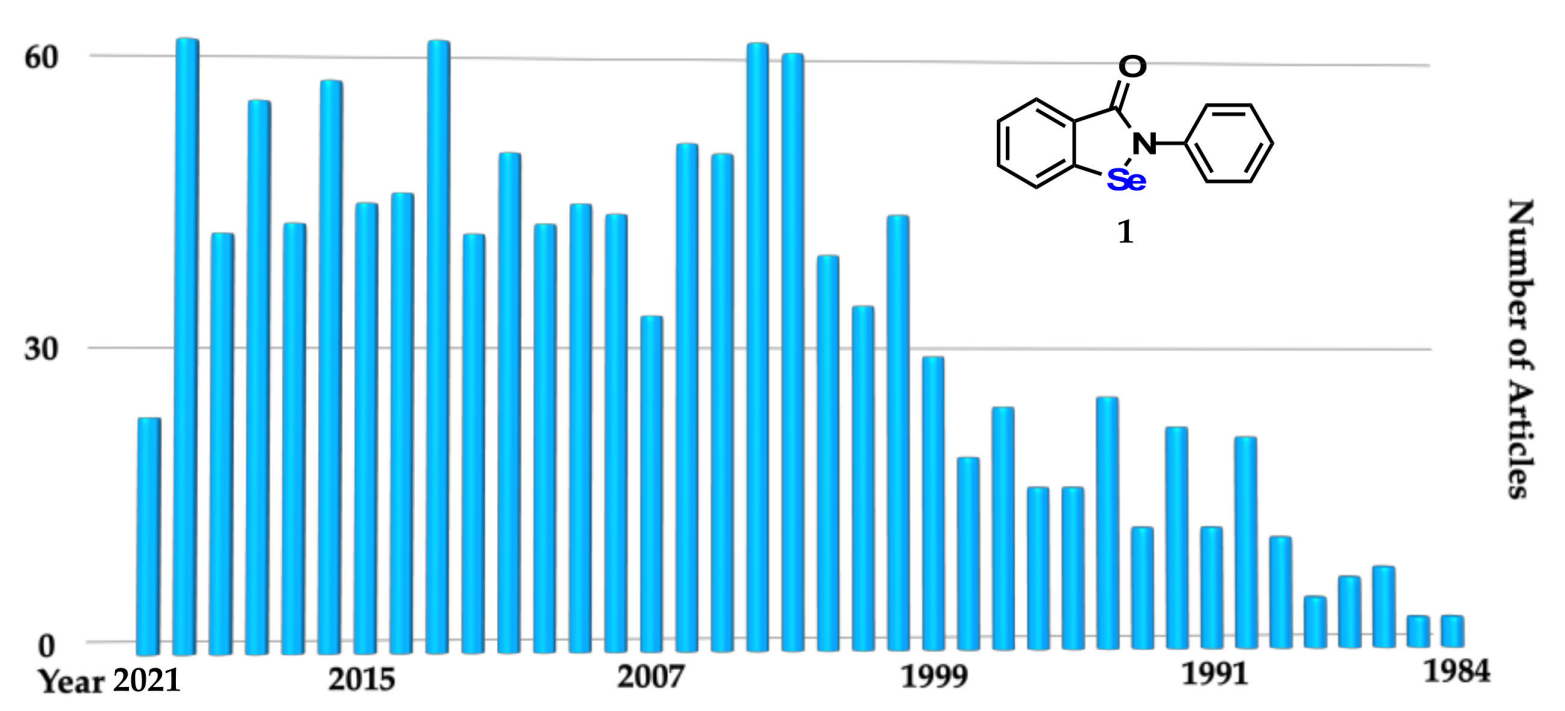
Number of articles on ebselen (**1**) from 1984.

2-Phenylbenzo[*d*][1,2]-selenazol-3(2*H*)-one (compound **1**, Figure 1), known as SPI-1005, DR3305, PZ-51 or ebselen, was synthesized the very first time by Lesser and Weiss in 1924 [1] in the context of a general organic chemistry paper. Exactly 60 years later, Helmuth Sies tested ebselen for its ability to mimic the antioxidant activity of the glutathione peroxidase (GPx) enzyme [2,3], which was recognized as a selenoenzyme few years before [4,5], paving the way for a completely new branch of investigation, the search for strategies and molecules able to cope with oxidative stress [6].

Since 1984, the research focused on ebselen spread incredibly with more that 1000 papers exponentially published. In Figure 1, a chart reports the number of articles plotted with respect to years. It was drawn by searching the term “ebselen” in PubMed.

Of course, this selection is devoid of the organic chemistry-oriented papers, which are excluded in PubMed. 

Ebselen was included in several clinical trials (see [7] for a list updated to 2017) both in US and Asia, but until now, no concrete perspective of clinical application is visible. Since 2020, due to it being highly active in inhibiting the SARS-CoV-2 main protease (see below), it has been under evaluation in a phase 2 study for its ability to combat SARS-CoV-2 infection in moderate as well as severely affected COVID-19 patients (https://clinicaltrials.gov/ct2/show/NCT04484025; https://clinicaltrials.gov/ct2/show/NCT04483973, accessed on 11 June 2021). 

In its long history, several biological activities were claimed for ebselen and several review articles [8,9] were published, some even recently [10,11]. One is particularly interesting because it places the selenorganic compound in the context of the SARS-CoV-2 treatment [12]. 

In this review we aim to collect and detail all the synthetic approaches reported for the preparation of such a valuable compound and its analogues; in addition, the results of their pharmacological evaluation, published after 2016, will be cited. Even if ebselen is known as an anti-inflammatory compound, the reader will not find a section entirely devoted to such property. The reason behind this choice is that no paper has yet been published precisely claiming such an activity; most probably because the anti-inflammatory property is so general that it is sometime masked or comprised in other, listed, pharmacological properties. 

## 2. Synthetic Approaches to Ebselen and Benzisoselenazolones

**Scheme 1:**
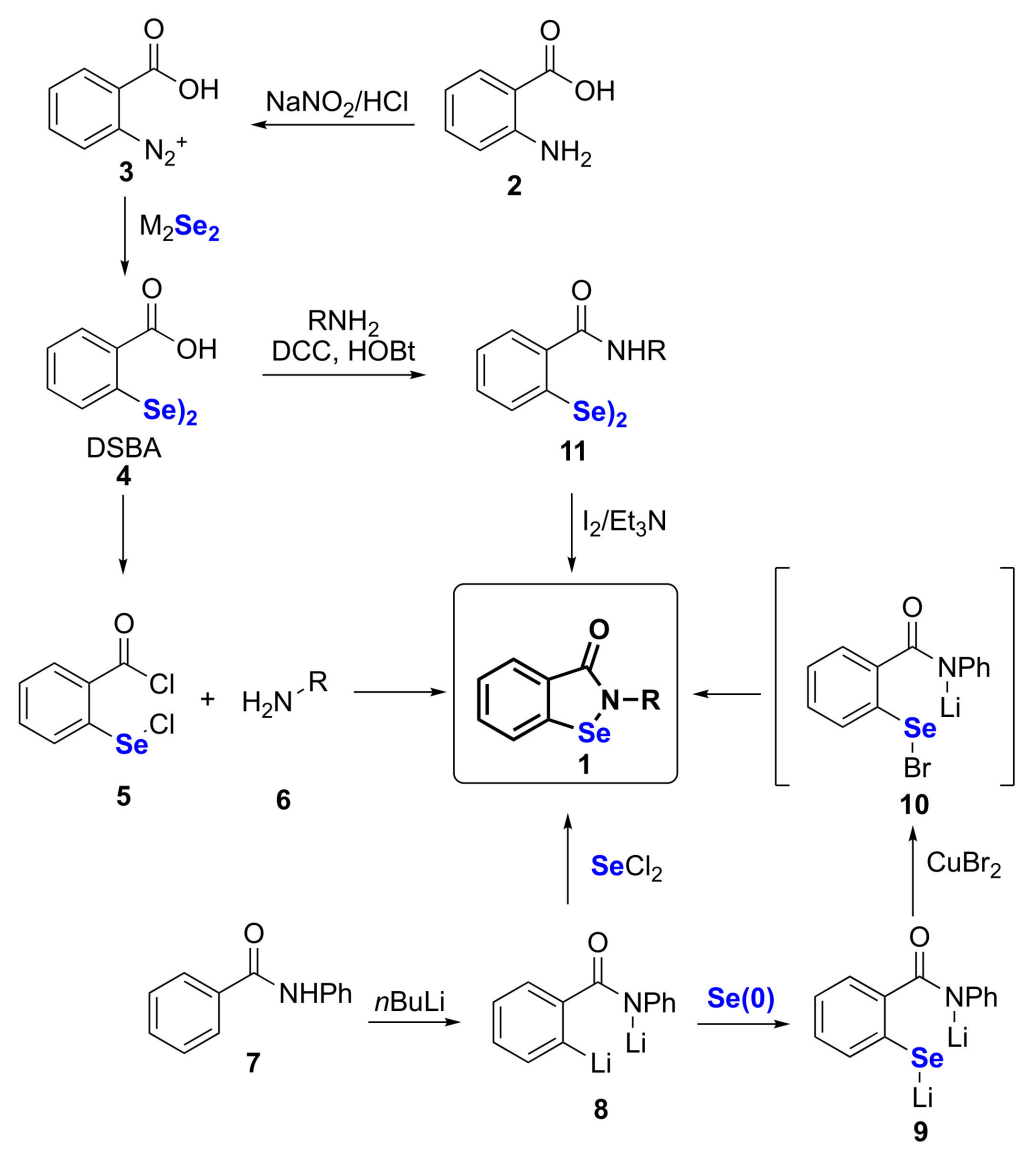
Synthetic approaches to **1**, part one.

As mentioned, ebselen was first prepared almost one hundred years ago by Lesser and Weiss even if, according to CAS SciFinder, the first report of benzisolenazolones synthesis can be traced back only to the 1980s [13,14], when it was built by reacting the dichloride **5**, plausibly prepared starting from the 2,2’-diselanediyldibenzoic acid (DSBA, **4**), with aniline. DSBA is another key intermediate for the synthesis of organoselenium compounds [15,16], endowed with notable biological properties [17]. Gütschow et al. adopted such a strategy to obtain fluorescent coumarin derivatives [18] while Pietka-Ottlik prepared benzisolenenazolones endowed with antimicrobial activity [19,20,21,22]. Similarly, Li et al. prepared a series of cholinesterase inhibitors by using anthranilic acid derivatives, proving the versatility of the synthetic protocol [23]. The reaction works also with terpenyl amines, giving chiral ebselen analogues [24] (Table 1, Method *1*). Engman in 1989 prepared the dianion **8** from N-phenylbenzamide **7** through ortho lithiation with nBuLi. Elemental selenium then inserts into the C-Li bond, affording the lithium selenide **9**, which is finally cyclized to ebselen using CuBr_2_, probably through the formation of the selenylbromide **10** [25]. This synthetic path was lately pursued by Silks to prepare the ^77^Se labeled version of ebselen [26] and recently adopted by Hsu for the preparation of a small benzisoselenazolones library, then assayed as radical scavengers [27] (Table 1, Method *2*). The direct conversion of dianion **8** into **1** through the treatment with the electrophilic SeCl_2_ was demonstrated by Singh in 2005 (Scheme 1) [28] (Table 1, Method *3*). 

The mixed anhydride strategy permitted us to convert DSBA into a set of diselenobisbenzamides **11** endowed with anti-HIV activity [29] due to their ability to inhibit the key viral protein NCp7 [30,31]. Compounds **11** were straight away cyclized into their ebselen-like analogues by the reaction with molecular iodine under basic activation [32]. Thionyl chloride, sulfuryl chloride or bromine are able to do the same job, as demonstrated by Christiaens, even if with longer reaction time [33] (Table 1, Method *4*).

**Table 1:** Synthetic procedures for ebselen **1** and its derivatives.

Method	Starting Compound	Number of Steps	Yield (%)	Ref
*1*	**2**	4	16–67	[18,19,20,21,22,23,24]
*2*	**7**	3	63	[25,26,27]
*3*	**7**	2	40	[28]
*4*	**2**	4	60	[15,16,32,33]
*5*	**13**	1	85	[34]
*6*	**12**	2	53	[35]
*7*	**15**	1	47–96	[36,37]
*8*	**15**	1	35–88	[38]
*9*	**15**	1	44–91	[39]
*10*	**15**	1	59–98	[40,41]
*11*	**16**	3	76	[42]

**Scheme 2:**
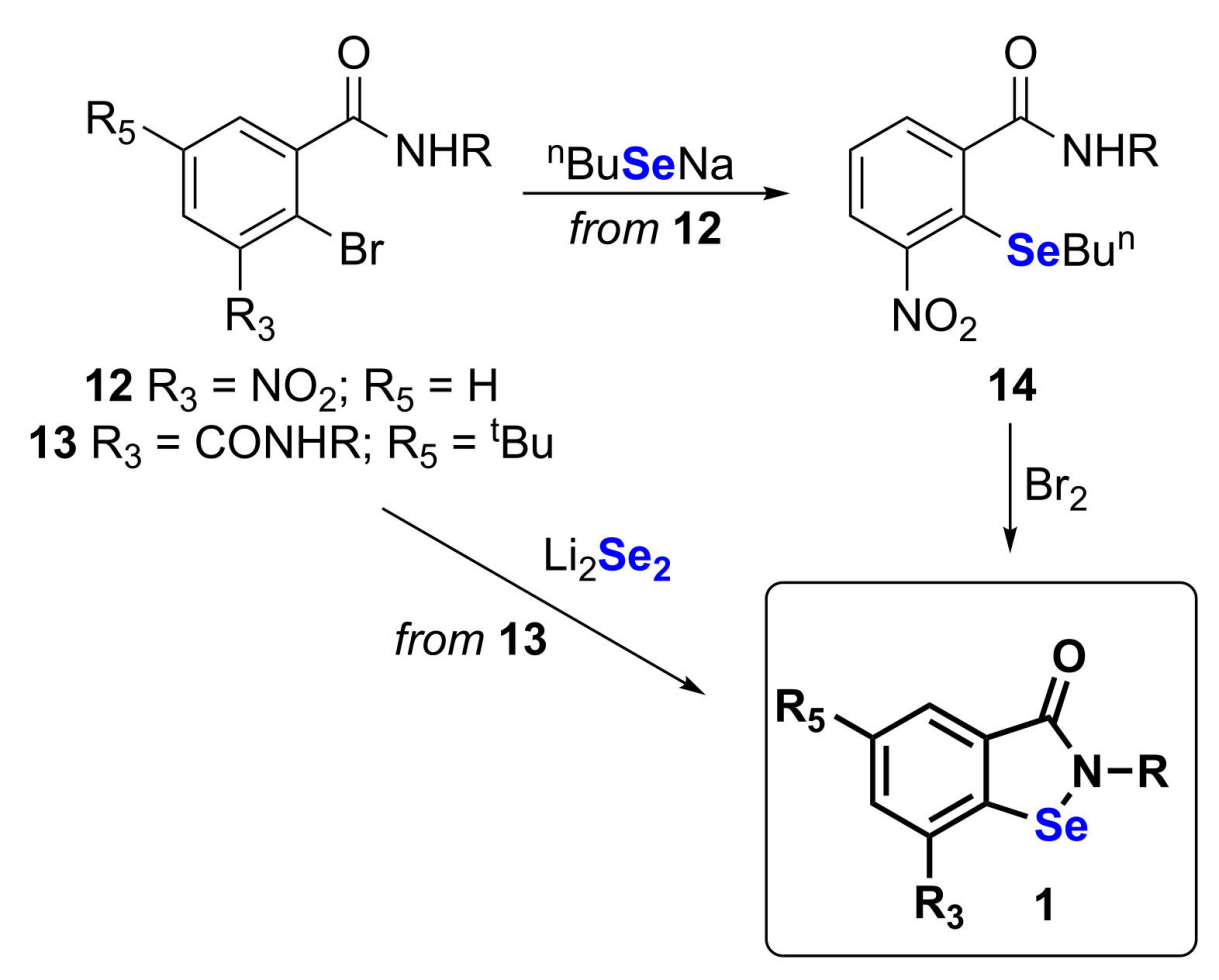
Synthetic approaches to **1**, part two.

In Scheme 2, the synthetic methodologies starting from *ortho*-bromo-benzamides are reported. In particular, the treatment of **13** with lithium diselenide in THF at reflux did not afford the expected diselenides but the ebselen analogue, instead [34] (Table 1, Method *5*). Very recently, Pal Sing and Engman prepared *ortho* nitro benzisolenazolones by the bromine-induced cyclization of **14**, in turn obtained by the aromatic nucleophilic substitution of **12** with sodium nButyl selenide [35] (Table 1, Method *6*).

**Scheme 3:**
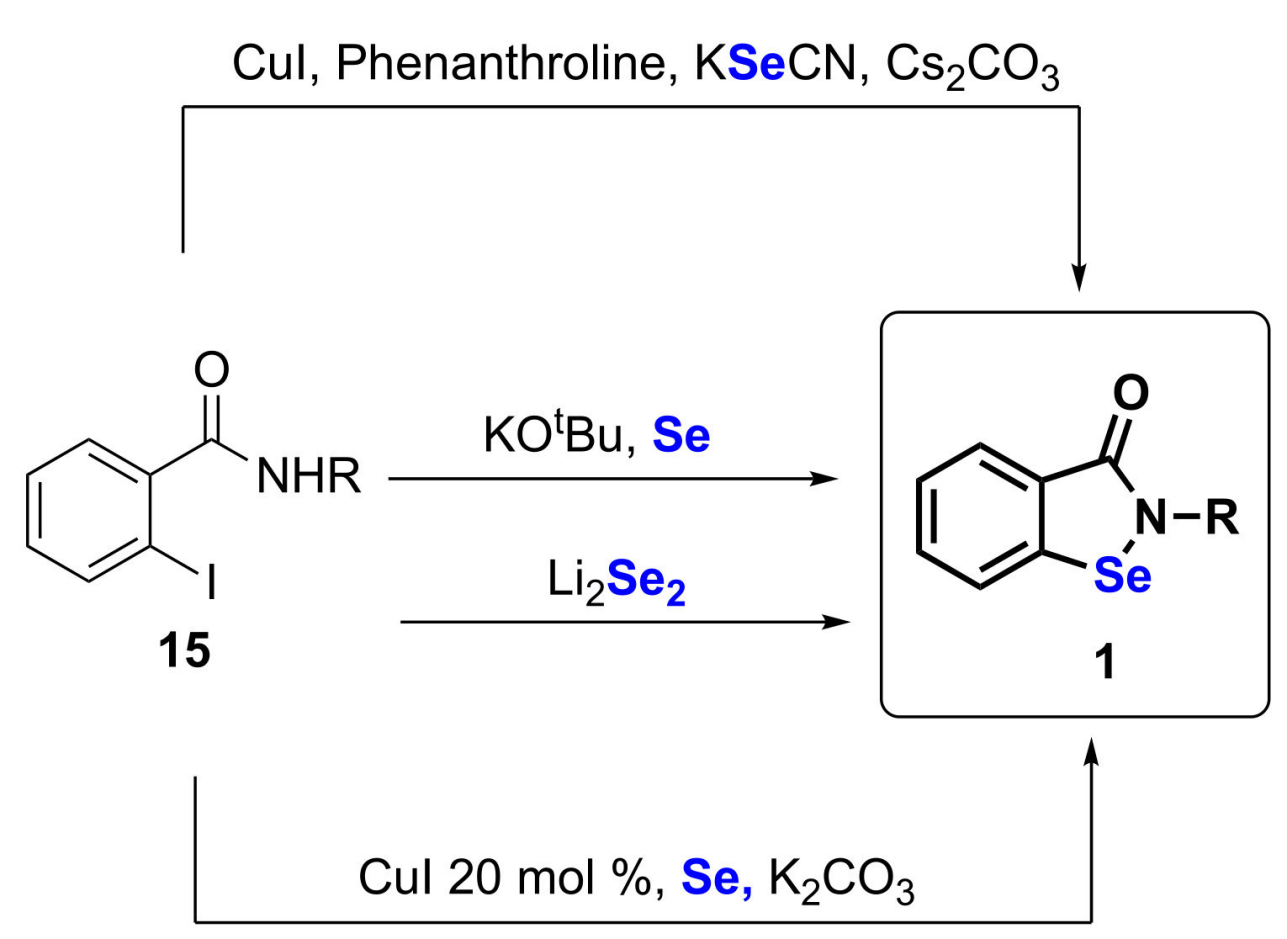
Synthetic approaches to **1**, part three.

*Ortho*-iodo-benzamides **15** were used as starting material for a number of approaches summarized in Scheme 3. A copper catalyzed approach was reported in 2010 by Kumar and coworkers, who used elemental selenium as selenium sources through what it is thought to be a double selenium insertion, the first one into the copper nitrogen complex and the second in the carbon iodide bond [36,37] (Table 1, Method *7*). The same authors later investigated the potassium *tert*-butoxide-promoted direct organication of elemental selenium [38] (Table 1, Method *8*). Stoichiometric amounts of copper iodide were necessary for the construction of the ebselen core in the study published by Sucheck and coworkers, who employed potassium selenocyanate as a selenium source. Interestingly the reaction works well under thermal and photo activation; in the latter case, phenanthroline is not required [39] (Table 1, Method *9*). Lithium diselenide in polar solvents is the selenium source in the protocol invented by Scianowski for the construction of diversely *N*-alkyl, *N*-aryl functionalized derivatives [40,41] (Table 1, Method *10*).

**Scheme 4:**
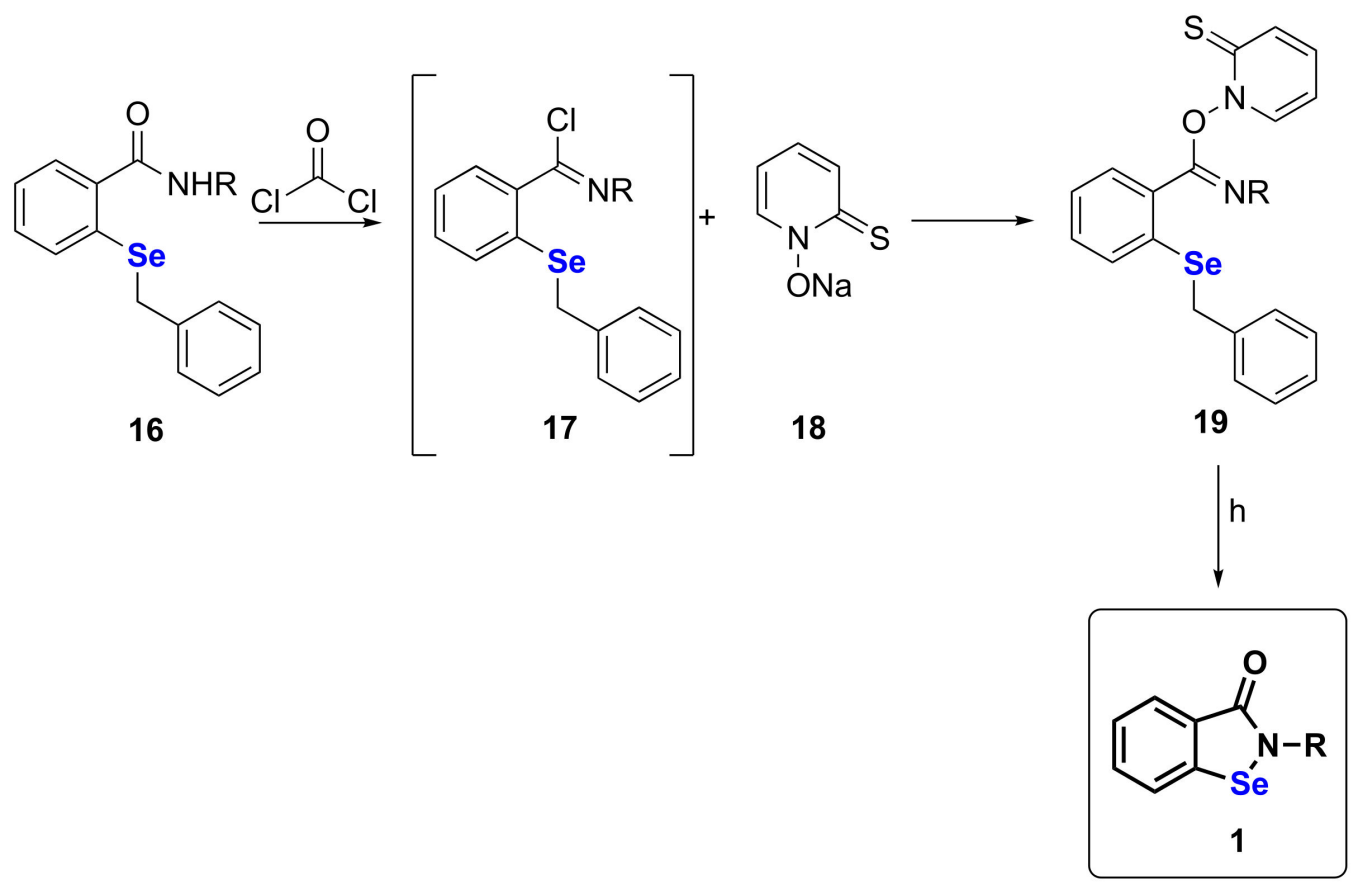
Synthetic approaches to **1**, part four.

Schiesser published the sole approach based on radical chemistry. In particular, the benzyl selenide **16** obtained by treatment of **8** with benzylbromide (not shown), was initially converted into the corresponding iminyl chloride **17** by the reaction with phosgene. Compound **17** was not isolated but reacted with the sodium salt of N-hydroxypyridine-2-thione **18,** leading to the pyridine-2-thioneoxycarbonyl imidate ester **19,** which readily underwent photolysis and radical ring closing reaction to afford ebselen (Scheme 4) [42] (Table 1, Method *11*). Recently, a review article by some of us gathered the synthetic efforts needed for the preparation of not just ebselen-like structures but also compounds bearing different Se-containing functionalities [43]. 

In Table 1, a summary of all of the synthetic procedures listed in this section is presented in order to help readers in recognizing the strategy more suited to their needs.

A useful tool to recognize ebselen, as well as the other selenorganic compounds, is ^77^Se NMR spectroscopy. Benzisoselenazolones display a characteristic chemical shift in a range of 900–960 ppm, depending on the used solvent [44].

## 3. Antimicrobial Activities

Several antimicrobial activities were reported for ebselen. Here they will be grouped as follows: antiviral, with particular emphasis on the anti-SARS-CoV-2 activity; antibacterial, effective against anti-mycobacterium tuberculosis; antifungal; and effective against other pathogenic microorganisms. Where reported, ebselen analogues will be detailed underlining the structural determinants that endow the molecules with a better or worse pharmacological profile. 

### 3.1. Antiviral

#### 3.1.1. Anti-Sars-CoV-2

In 2019, a tremendous viral infection started in China and spread all over the world, revolutionizing our everyday life. The virus was identified as a coronavirus of zoonotic origin, named severe acute respiratory syndrome coronavirus 2 (SARS-CoV-2), which is recognized as the etiological agent responsible for the 2019–2021 viral pneumonia named COVID-19 [45]. With their pivotal paper, Yang and coworkers solved the crystal structure of the key viral enzyme main protease (M^pro^) in complex with the peptidomimetic inhibitor N3. In the frame of a drug repurposing approach, they tested the so-called “clinical collection”, a library of compounds with a history of clinical trials, identifying ebselen as the most potent M^pro^ inhibitor with an IC_50_ of 0.67 μM. Interestingly, ebselen covalently inhibits the enzyme, being nucleophilically attached by Cys145 within the enzyme active site. The selenylsulfide thus formed is stable enough to permanently inhibit enzymatic activity, resulting in viral inhibition, as demonstrated also in a cellular model of viral replication where ebselen showed an EC_50_ of 4.67 μM [46]. This discovery made the repurposing of ebselen for SARS-CoV-2 treatment a reasonable option, so much so that it entered a phase II clinical trial [47].

Interestingly, the reliability of ebselen as a clinically exploitable M^pro^ inhibitor was questioned by Wang et al., who investigated the specificity of the covalent M^pro^ inhibitors known at that time, demonstrating that the inhibition is fully reversible by the addition of reducing agents such as dithiotreithiol or GSH [48]. 

The M^pro^ binding capabilities of ebselen were thoroughly investigated computationally, suggesting the possibility to exploit the benzisoselenazolone core not only as an active site binder but also as an allosteric modulator [49]. Very recently the studies regarding the interaction between ebselen and Sars-CoV-2 proteases were extended to the putative ebselen metabolites [50]. 

Ebselen was later found to inhibit the Zn-containing non-structural proteins (Nsp) 13 and 14. Nsp13, which is an helicasetriphosphatase pivotal for the capping of nascent viral mRNAs, is inhibited with an IC_50_ of 292 nM, while the exoribonuclease activity of nsp14 is blocked with an IC_50_ potency of 3.18 μM. The same paper demonstrated that the compound displays synergism with the newly approved anti-SARS-CoV-2 drug Remdesivir [51]. 

**Figure 2:**
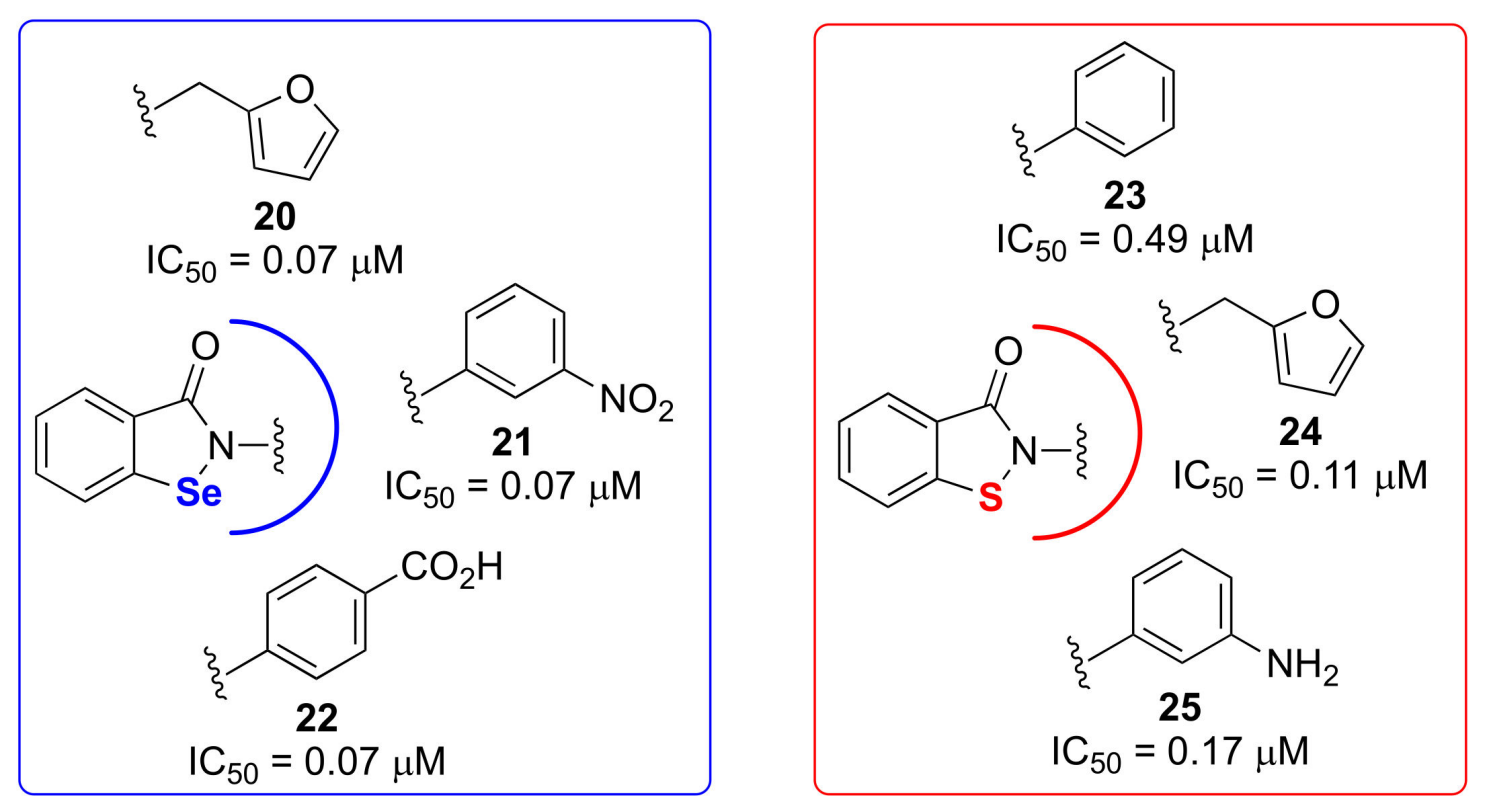
Ebselen and ebsulfur analogues endowed with anti-M^pro^ activity.

Inspired by these results, several research groups engaged in drug discovery campaigns; even if some preprints are reported, only the peer reviews papers will be detailed. Weglarz-Tomczak et al. reported a series of ebselen-like structures able to inhibit not only M^pro^, but also the other pivotal protease, a papain-like protease from SARS-CoV-2 with comparable potencies, in the high micromolar range [52]. Yang et al. prepared a series of ebselen and ebsulfur (compound **23**, Figure 2) analogues variously functionalized at the nitrogen atom with aryl, alkyl, benzyl and heteroaryl substituent identifying compounds endowed with a better M^pro^ activity when compared to the native lead compound; interestingly, not only ebselen but also **23** proved to covalently bind M^pro^ [53]. It is worth mentioning that ebselen-like structures are always more potent when compared to their sulfur analogues.

**Scheme 5:**
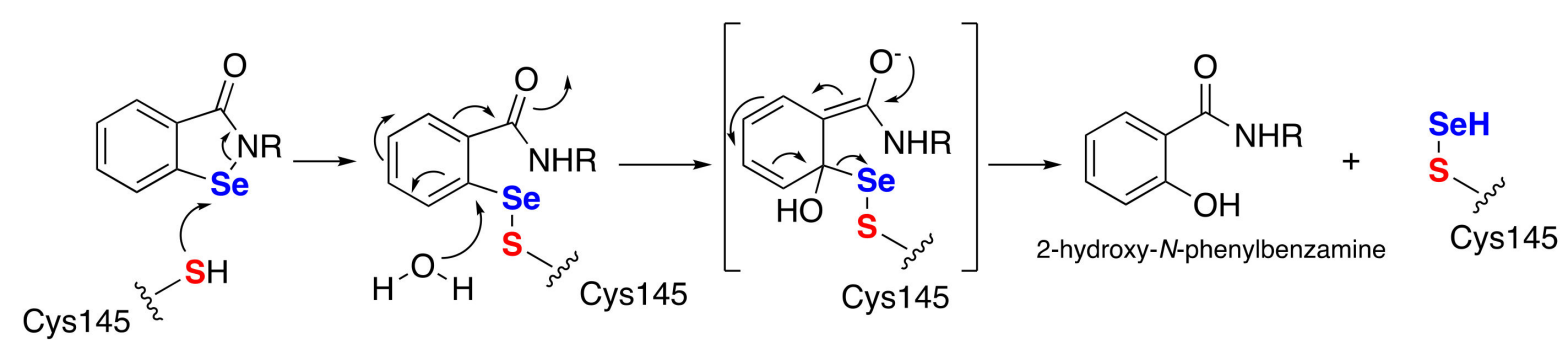
Selenenylation of M^pro^ by ebselen-like compounds.

At the time the present review article was almost complete, a very important paper was published by Yang and coworkers who, in an attempt to obtain co-crystal structures between ebselen, its analogues and M^pro^, found that after protein/small molecule incubation only the selenium atom is present in the enzyme active site, suggesting the mechanism summarized in Scheme 5. In particular, once the selenenyl sulfide intermediate is formed, it reacts through a SNAr-like mechanism with water, leading to the formation of 2-hydroxy-*N*-phenylbenzamide and the selenenylated Cys145 [54]. 

#### 3.1.2. Anti-HIV

Despite remarkable progress in treating the human immunodeficiency virus (HIV) the causative agents of the acquired immunodeficiency syndrome are still a serious treat worldwide and much needs to be done to achieve its eradication [55]. In the past few years ebselen was reported as a valuable anti-HIV compound because of its multiple activities. It was indeed capable of inhibiting the HIV capsid protein dimerization with activity in the low nanomolar range, by covalently linking at Cys198 and Cys218 of the capsid C-terminal domain, as proved by NMR analysis and electrospray ionization mass spectrometry. When tested in a cellular context, ebselen presented antiviral properties in single and multiple rounds of infection in permissive cell lines as well as in primary peripheral blood mononuclear cells [56].

Later on, Luo et al. proposed ebselen as an indirect integrase inhibitor thanks to its ability to bind to lens epithelium-derived growth factor (LEDGF/p75), a cellular factor hijacked by the HIV-1 virus to allow itself a proper integration process. In this case, the interaction is also thought to be covalent, even if no direct proof was given [57].

#### 3.1.3. Anti-Influenza, Zika and Other Viruses

Influenza viruses (flu), are important pathogens responsible for yearly seasonal epidemics and more extensive global pandemics. Indeed, pandemic outbreaks could occur in the case that highly virulent and pathogenic viral strains, deriving from antigenic shift, emerge. Such viral infections are a common cause of acute exacerbations of chronic obstructive pulmonary disease, which is among the major leading causes of death worldwide. This scenario becomes worse after exposure to cigarette smoke, which increases the oxidative stress within the respiratory tract and disrupts the resolution of influenza infection in vivo [58]. Due to its ability to reduce lung inflammation triggered by several stimuli [59,60] and being the prototypical antioxidant, ebselen was tested in an animal model of chronic obstructive pulmonary disease in which both the influenza virus infection and the damage triggered by cigarettes smoke were simulated. It was administered orally and lowered viral titers, when compared with the control group, by reducing viral replication at the very early stage of infection; in addition it reduced oxidative stress and the expression of pro-inflammatory mediators in the lung [61]. 

Interestingly, very recently ebselen proved to reduce the endothelial disfunction induced by the smoke from cigarettes in vivo [62].

Zika virus (ZIKV) belongs to the *flaviviridae* family, and is a single-stranded RNA virus that recently created a public health emergency of international concern [63]. Even if its infection has been associated with low cases of fatality, it causes microcephaly in infants and Guillain-Barre syndrome. To date there is no viable or approved treatment, thus the prevention of ZIKV transmission is the sole strategy for disease control and management [64]. Even if the ZIKV transmission prevalently requires an intermediate mosquito (Aedes) host, it was proven that sexual transmission can be an additional and even more dangerous route. Such viral infection creates testicular oxidative stress and pro-inflammatory response, either impairing the male reproductive capabilities or improving the transmissibility of the virus itself [65]. In 2018, Lin et al. proposed the use of ebselen as a possible therapeutic intervention to alleviate the testicular pathogenesis and prevent the sexual transmission of ZIKV. The selenorganic compound displayed a poor effect on ZIKV replication in culture cells but when administered intraperitoneally at 10 mg/Kg dose it was able to prevent ZIKV sexual transmission and improved testicular oxidative stress by altering the cytokine profile of treated mice [66]. 

In 2017, in the frame of a wider study, ebselen together with two of its close analogues were prepared and tested against human herpes virus type 1 (HHV-1), encephalomyocarditis virus (EMCV) and vesicular stomatitis virus (VSV), replicating in the human cell line A549. With the exception of VSV, ebselen and analogues displayed promising activity in terms of minimum inhibitory concentration (MIC), which was in the range of μg/mL [67].

### 3.2. Antibacterial

#### 3.2.1. Against Pathogenetic Gram-Positive Bacteria

**Figure 3:**
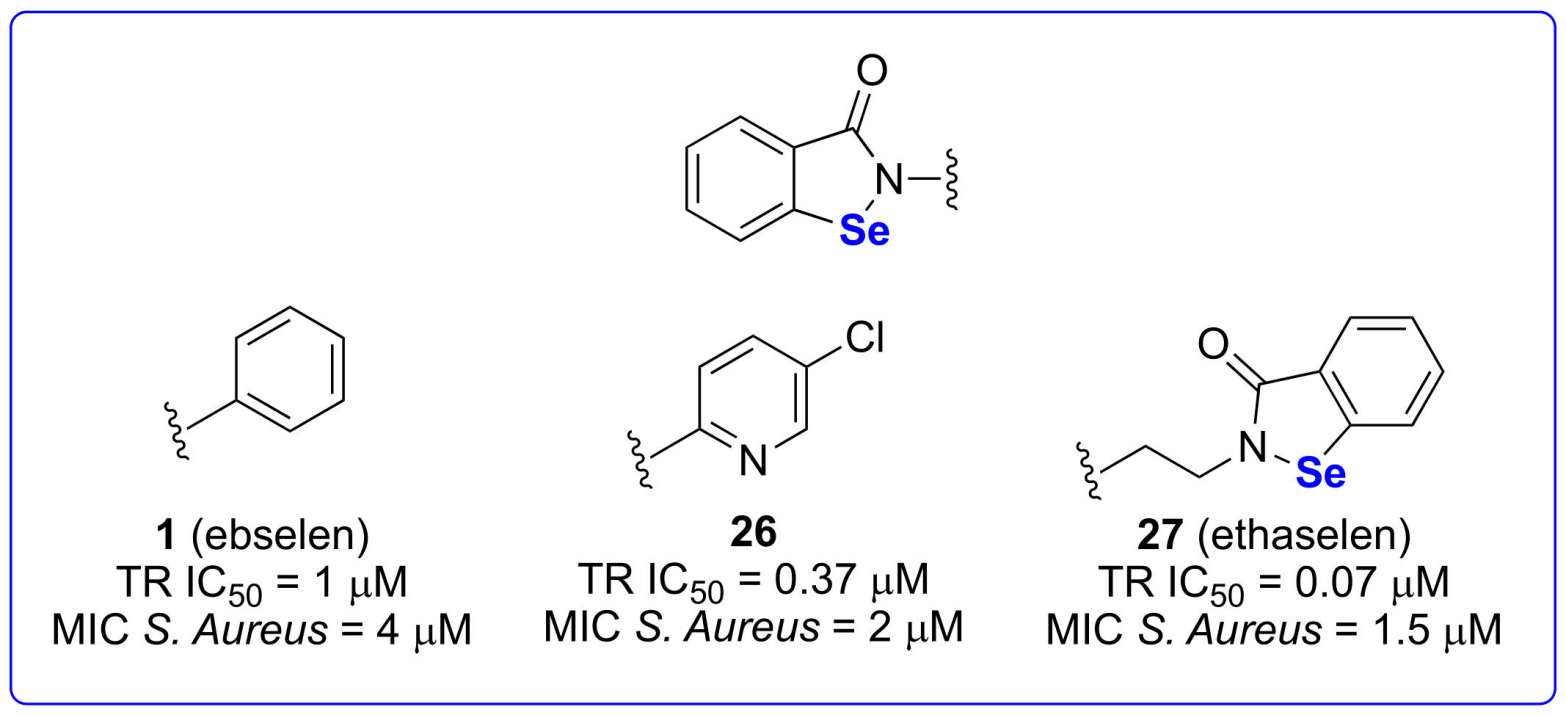
Antibacterial TR inhibitors.

Some Gram-positive bacteria, such as *Staphylococcus aureus*, *Bacillus anthracis, Clostridium difficile, Enterococcus faecium* and *Enterococcus faecalis,* are pathogenic microorganisms able to cause serious diseases upon infection. *B. anthracis* causes anthrax, which is associated with a very high mortality rate, and it is perfectly shaped from a bioterrorist point of view. Notably, problems with methicillin-resistant (MRSA) and multidrug-resistant (MDRSA) *S. aureus* are becoming more and more common; thus the development of new antibacterial agents endowed with a mechanism of action different from that of the clinically used drugs is an urgent need. The thioredoxin system was selected as a target by Holmgren and coworkers in their drug discovery campaign meant to discover agents capable of inhibiting the growth of *B. anthracis* and/or MRSA. Thirteen derivatives were prepared, among them the 4-chloro-pyridin-2-yl-containing compound **26** and the dimer ethaselen **27** are worth mentioning (Figure 3). They displayed a higher potency in inhibiting thioredoxin reductase (TR) when compared to ebselen and showed a potent MIC against both *S. aureus* and *B. anthracis* [68].

Very recently, ebselen was proved not only to inhibit *S. aureus* TR but also to sensitize the enzyme to curcumin, improving its activity to block the enzyme. In the frame of the same investigation, ebselen was tested topically in a rat model of staphylococcal skin wound infection, proving to have high therapeutic efficacy against MDRSA [69]. 

Seleem et al. proved that ebselen was unable to select *S. aureus*-resistant variants even after 14 consecutive passages [70]. Pietka-Ottlik prepared ebselen, a series of its analogues and a small library of diselenides, then tested for their ability to inhibit *S.aureus* and other Gram-positive pathogenic bacteria, identifying some suitable preclinical candidates [67]. 

Pressure ulcers (PUs) are a serious threat worldwide, affecting people with limited mobility, including those who are obese, and their bacterial infection surely represents a leading cause of hospital admissions. *S. aureus* is among the most dangerous bacterial species that often infect PUs. For this reason, ebselen was tested topically for its ability to reduce the burden of MRSA in infected PUs both in obese and diabetic mice but, disappointingly, it proved to be ineffective [71]. The same authors proposed ebselen as a valuable agent to decolonize vancomycin-resistant enterococci (VRE) from the gastrointestinal tract (GIT) of high-risk patients. Among the most problematic enterococci, *Enterococcus faecium* and *Enterococcus faecalis* are listed as high-priority pathogens by the World Health Organization (WHO). Within their study, the antibacterial activity of ebselen, together with clinically used antibacterial drugs, was evaluated against several strains of enterococci from humans and animals, revealing that the selenorganic compound was found to have antibacterial activity against all the tested isolates, whereas the control compound lacked activity against some strains. In the resistance selection test, ebselen was evaluated via a multi-step resistance selection experiment against vancomycin-resistant *E. faecium*, with the MIC unchanged after 14 passages. In contrast, resistance to gentamicin, which was tested in parallel, emerged rapidly. The antibiofilm capabilities were also determined, and it proved to be superior when compared to all the other tested drugs in eradicating preformed VRE biofilms [70]. This finding is really important; indeed, very few examples of selenorganic compounds endowed with such an activity are reported in literature [72]. 

Ebselen was then tested in vivo in mice colonized with VRE. It was administered orally at 10 mg/Kg dose and it was able to significantly reduce the bacterial burden in fecal samples after just three days of treatment [70].

*Clostridium difficile* infection (CDI) is directly linked to thousands of deaths per year in both the US and in Europe [73]. CDI is facilitated by a wrong use of antibiotics, which induces dysbiosis in the commensal microbial communities of the GIT. A number of commercially available antibacterial drugs taken for unrelated conditions have been shown to disrupt the GIT microbiome in humans, causing a state that is permissive for CDI [74]. Ebselen was reported to act as valuable anti-CDI agent in vivo by covalently inhibiting large clostridial toxins ((toxins A (TcdA) and B (TcdB)) [75]. Besides, the selenorganic compound proved not only to block the onset of CDI but also to reduce the recurrence rates of CDI, decreasing at the same time colitis in a hamster model [76]. 

#### 3.2.2. Against Pathogenetic Gram-Negative Bacteria

**Figure 4:**
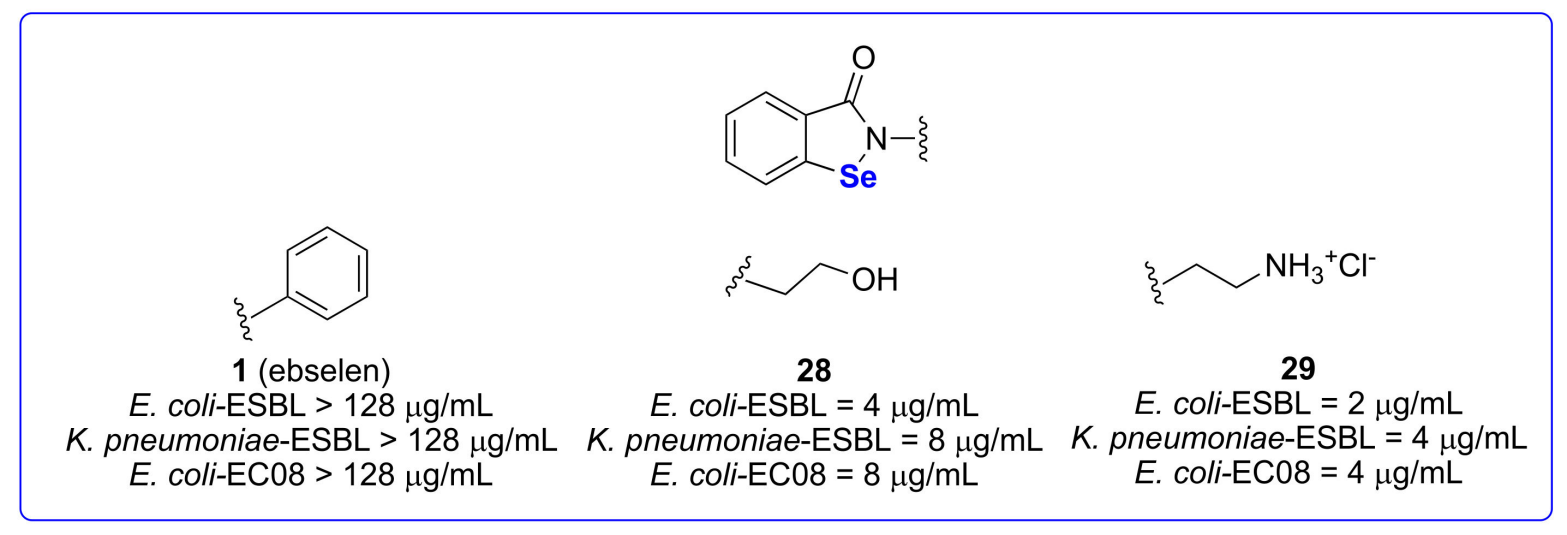
Hydrophilic ebselen analogues endowed with anti-Gram-negative bacteria properties.

The increasing occurrence of MDR Gram-negative bacteria has become a tremendous health threat worldwide. *Klebsiella pneumoniae*, *Pseudomonas aeruginosa*, *Acinetobacter baumannii* and *Escherichia coli* are just a selection of what the WHO defines as “priority pathogens” [77]. The main difference between Gram-positive and Gram-negative bacteria is that the latter have two cellular membranes, with the outer one being impermeable to most small molecules. This natural barrier poses a tremendous challenge to the medicinal chemistry community that tries to develop broad-spectrum antibacterial agents [78]. Among the strategies to allow a better penetration into the periplasm, the increment of the hydrophilicity is among the most pursued. With this in mind, Yang and Chen prepared a series of ebselen analogues bearing aliphatic and polar functionality at the amidic nitrogen (representative compounds **28** and **29**, Figure 4) [79].

Compounds **28** and **29**, being way more hydrophilic than ebselen, readily accumulated into the Gram-negative pathogenic bacteria, most probably by passing through the porins and exerted a powerful bactericidal activity with potency in the low micromolar range [79]. The *K. pneumonia* tested in this study expresses the so-called New Delhi metallo-β-lactamase (NDM-1), a kind of metallo-β-lactamases capable of destroying β-lactam-containing antibiotics by attacking the carbonyl group of the β-lactam through a zinc-bound hydroxyl ion. Such an enzyme was the target of a drug discovery process very recently carried out by Chan et al., who employed the benzisoselenazolone and its sulfur analogue benzisothiazole cores as templates for the synthesis of potent NDM-1 blockers [80,81,82].

In 2017, Holmgren et al. reported a synergistic activity between compound **1** and silver ions in inhibiting both *E. coli* thioredoxin and TR, highlighting that TR could be a suitable target not only in the anti-Gram-positive bacteria research field. The combination of the selenorganic compound and the metal increased the reactive oxygen species (ROS) content, lowering the MIC of the metal and making it more selectively toxic to bacteria over mammalian cells [83]. In 2020, the very same concept was implemented by Zou and colleagues who, using a clinically isolated uropathogenic *E. coli* strain, proved the efficiency of an ebselen/silver ion combination in reducing acute cystitis infections in a mouse model [84]. 

**Figure 5:**
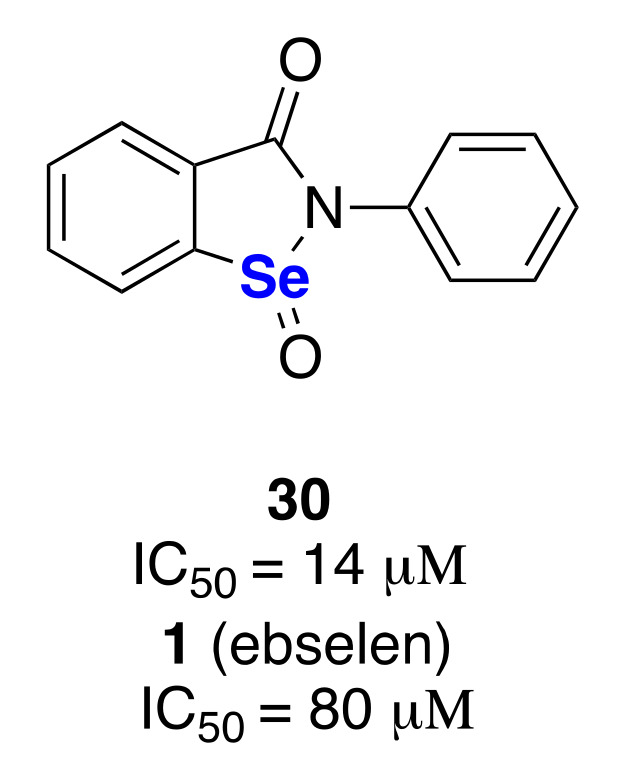
Structure of IC_50_ for alginate inhibition.

*P. aeruginosa* is an opportunistic pathogen that causes acute as well as chronic infections in immunodeficient people, such as those under antineoplastic chemotherapy or AIDS patients [85]. Importantly, *P. aeruginosa* is found in the airways of cystic fibrosis (CF) patients, whose mucociliary clearance is hampered, making the airways more susceptible to microbe infections [86]. In the context of CF, *P. aeruginosa* is able to develop a particular resistance mechanism. It starts to produce alginate by overexpressing alginate biosynthesis proteins, building a physical shield and making it resistant to antibiotics and, at the same time, further occluding and impairing lung function [87]. Very recently, Lee et al. discovered ebselen-like compounds and ebselen selenoxide (compound **30**, Figure 5) as valuable anti-Pseudomonas agents capable of inhibiting alginate production. Interestingly the selenoxide derivative showed a sixfold higher potency, if compared to **1** [88].

#### 3.2.3. Against Mycobacterium Tuberculosis

**Figure 6:**
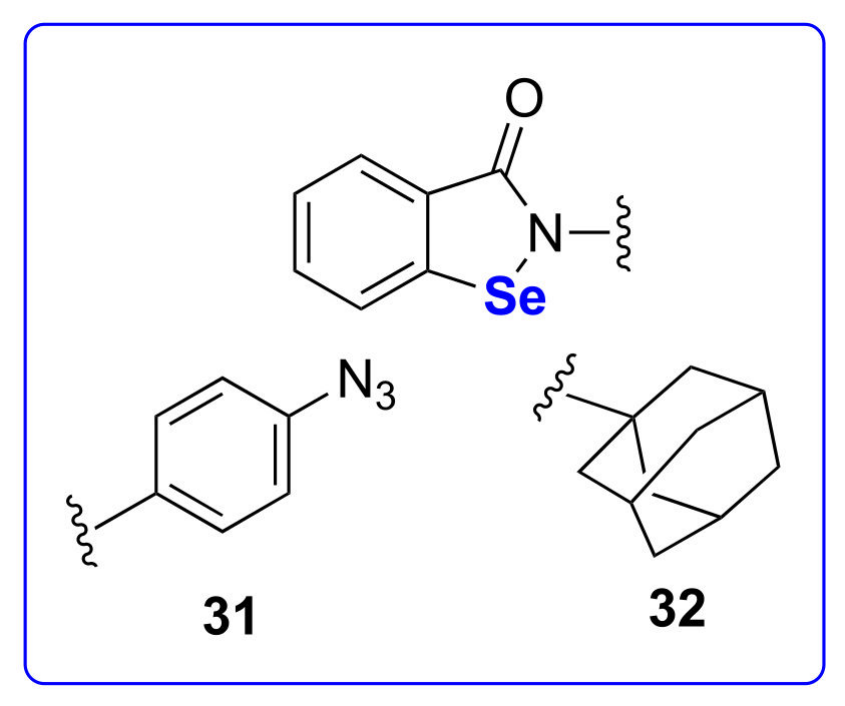
Structures of *Mtb* Ag85C binders.

Tuberculosis is an old infectious disease caused by *Mycobacterium tuberculosis (Mbt),* a kind of microorganism that represents the transition state between fungi and bacteria, falling into both the Gram-positive and negative clusters. The standard of care relies on two lines of intervention which differ according to the tolerability of the drug treatment but, in both cases, last for months. Most of the anti-*Mbt* first-line drugs are poorly tolerated and as a result the adherence sometimes fails, making the selection of multidrug- and total drug-resistant strains a common outcome [89]. For this reason, the development of newer anti-*Mbt* drugs is highly demanded. In 2013, ebselen was discovered as a potent inhibitor of the three essential homologous *Mtb* proteins of the antigen 85 complex (Ag85A, Ag85B, andAg85C) and having a MIC of 20 μg/mL [90]. Starting from this milestone, a series of ebselen analogues was designed and synthesized [39] and in a recent report, two highly promising derivatives (compound **31** bearing a 4-azido-phenyl moiety and **32** bearing an adamantly functionality, Figure 6) were co-crystallized in the presence of *Mtb* Ag85C, yielding two X-ray crystal structures of 2.01 and 1.30 Å resolution, that surely represent a valuable tool for the design of more potent analogues. As expected, both structures displayed the anticipated covalent modification of the noncatalytic Cys209 residue, forming a selenenylsulfide bond [91]. 

The L,D-transpeptidase of *Mbt* is considered an innovative target, which is not currently reached by the used drugs, for the development of next-generation anti-mycobacterium drugs. It was the protein selected Brem et al., who discovered ebselen by screening a library of cysteine-reactive reagents. The selenorganic compound binds to Cys354, proving that such a protein could be targeted not just with β-lactamines [92].

#### 3.2.4. Against Pathogenetic Fungi

Among the most widespread infectious diseases globally, the ones caused by fungi are worth mentioning. Almost one billion cases of either superficial or cutaneous mycoses are recorded around the world per year and they account for 1.6 million global deaths [93]. In this scenario, the search for agents able to prevent or subvert mycoses is a global priority. Diphenyl diselenide and ebselen are among the most studied selenorganic compounds as antifungal agents and recently a review article focused on the medical aspects has been published [11], for this reason, herein the attention will be devoted to medicinal chemistry-oriented reports focused on ebselen and analogues even if examples of selenorganic compounds endowed with antifungal properties were reported [67,94].

In 2017, compound **1** was assayed against 15 strains of *Candida albicans*, 2 of *C. glabrata*, 2 of *C. tropicalis*, *C. parapsilosis* and 9 of *Cryptococcus neoformans* in vitro, and subsequently it was tested in vivo in a *Caenorhabditis elegans* animal model. Ebselen showed MICs ranging from 0.5 to 2 μg/mL and it proved to be better than the clinically used fluconazole, flucytosine and amphotericin in reducing *Candida* and *Cryptococcus* fungal load in vivo. From a mechanism of action point of view, ebselen was able to deplete fungal cells of glutathione (GSH), triggering a ROS storm that eventually kills the microbe [95]. 

**Figure 7:**
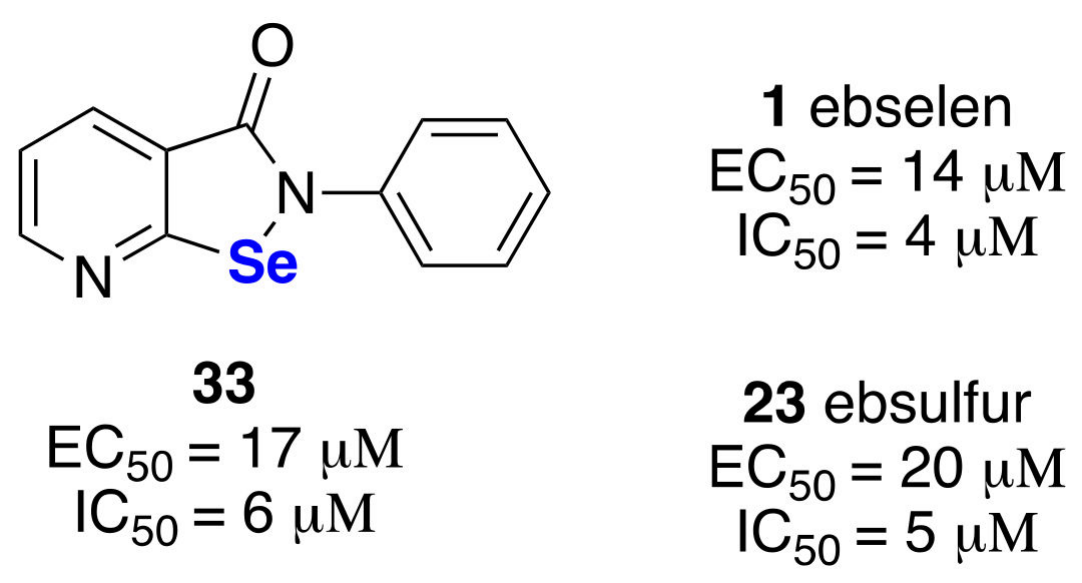
Structure of compound **32** and pharmacological data for compounds **1**, **23** and **33**.

The same year, Billack et al. reported a series of ebselen analogues as inhibitors of the plasma membrane H^+^-ATPase proton pump (Pma1p), which is considered an intriguing target since it is critical to fungal survival. Beside ebselen, which showed an interesting inhibitory activity toward the pump (IC_50_ = 4 μM) and the growth of fluconazole-resistant *C. albicans* strain, ebsulfur (compound **23**, Figure 2) and the aza-compound **33** (Figure 7) also showed promise [96]. The same authors recently developed a nanoemulgel-based formulation of ebselen for the treatment of superficial candidiasis, which showed a higher activity when compared with terbinafine even at high concentration [97]. 

*Candida auris*, isolated for the first time in 2009, is now considered an emerging pathogen since, from that time, it quickly spread and has become a growing threat in hospitals around the globe [98]. Unfortunately, antifungal therapy against infections caused by *C. auris* is ineffective because up to 90% of isolates are resistant to fluconazole and other azoles. In an attempt to discover suitable agents against this emerging pathogen, Lopez-Ribot et al. reported a drug repurposing approach in which they screened the Prestwick Chemical library, composed of 1200 small molecules with a history of clinical use. From this screening, ebselen emerged as the best-in-class compound with an IC_50_ of 1.41 μM. In the frame of the same study, ebselen proved to be effective also against *C. auris* clinical isolates. The selenorganic compounds proved to be able to inhibit biofilm formation where the clinically used echinocandins proved to be inactive [99]. 

#### 3.2.5. Against Other Pathogenetic Microorganisms

**Figure 8:**
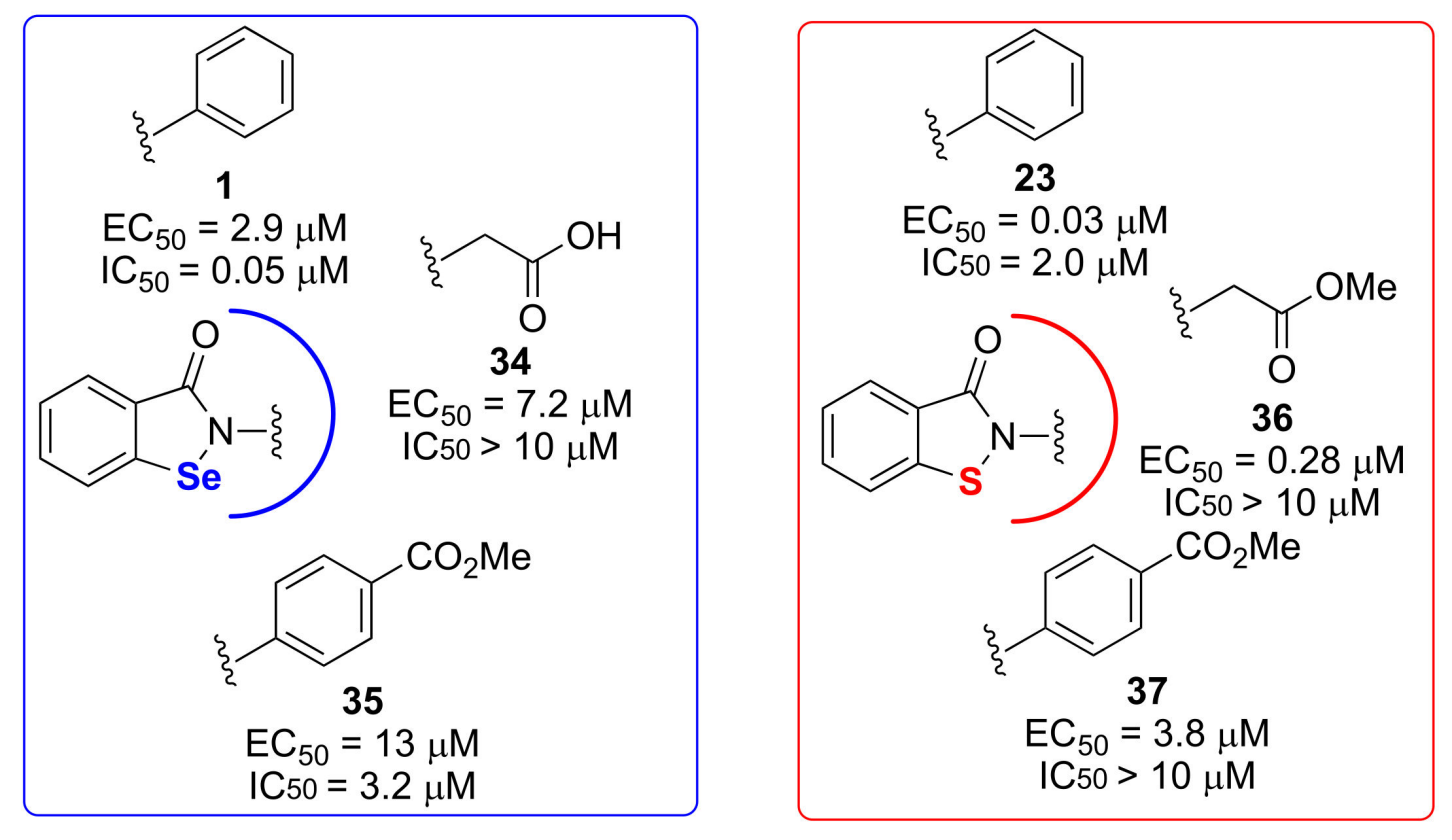
Anti-*T. brucei* compounds. EC_50_ is the ability of compounds to inhibit *T. brucei* growth while IC_50_ is the enzymatic activity.

African sleeping sickness, caused by the eukaryotic pathogen *Trypanosoma brucei,* is a serious problem, particularly in sub-Saharan Africa, with no apparent solution since the available treatments are largely ineffective or poorly tolerated [100]. Both Ebselen and its sulfur analogue, ebsulfur (compound **23**), were reported to exert anti-*T. brucei* activity by inhibiting its hexokinase 1 (TbHK1), which is a pivotal enzyme for the pathogen energy production chain [101]. Recently, the benzoisoselenazole and thiazole scaffolds were studied through the synthesis of analogues, then assayed for the inhibition of TbHK1 activity and *T. brucei* growth. The most interesting compounds are reported in Figure 8. Interestingly, the removal of the N-phenyl ring abolishes the anti-TbHK1 activity (compound **34** and **36**). Nevertheless, such compounds still have the ability to inhibit the trypanosome cellular growth with micromolar potencies [102].

*Cryptosporidium* parasites are the causative agents of severe diarrhea that impedes the growth of children and sometime is associated with premature child death [103]. Unfortunately, nitazoxanide is the only approved drug to treat cryptosporidiosis, but its use is limited to immunocompetent patients, while for immunocompromised ones there are no effective treatments for such a condition. In 2018, by testing the Prestwick Chemical library, ebselen was identified as an inhibitor of the glucose-6-phosphate isomerase of *C. parvum* (CpGPI). The enzymatic inhibitory activity translated to the ability to inhibit the growth of the parasite in vitro. Interestingly, the selenorganic compound proved to be more selective for the protozoal rather than the human isoform of the same enzyme [104].

Primary amebic meningoencephalitis is a rare and fatal disease of the central nervous system caused by the amoeba *Naegleria fowleri*. No effective treatments are available and the drug discovery projects are made difficult by the fact that the compounds need to cross the blood–brain barrier (BBB) to be active. Ebselen, and one of its close analogues, being known to cross the BBB, were tested and found active in killing *N. fowleri* trophozoites in vitro with an activity of 6.2 μM, which was notably lower than the concentration ebselen reaches in plasma, making its actual clinical exploitability valid as a ready-to-use alternative [105]. 

## 4. Antiproliferative Activities

According to the WHO, cancer is the second leading cause of death globally, with almost 10 million deaths in 2020 [106]. In recent years, efforts have been made to design and synthesize a number of Se-organic molecules in an attempt to develop new strategies for the treatment of neoplastic disorders. In this context, research papers and reviews have been published [107,108,109,110,111]. 

**Figure 9:**
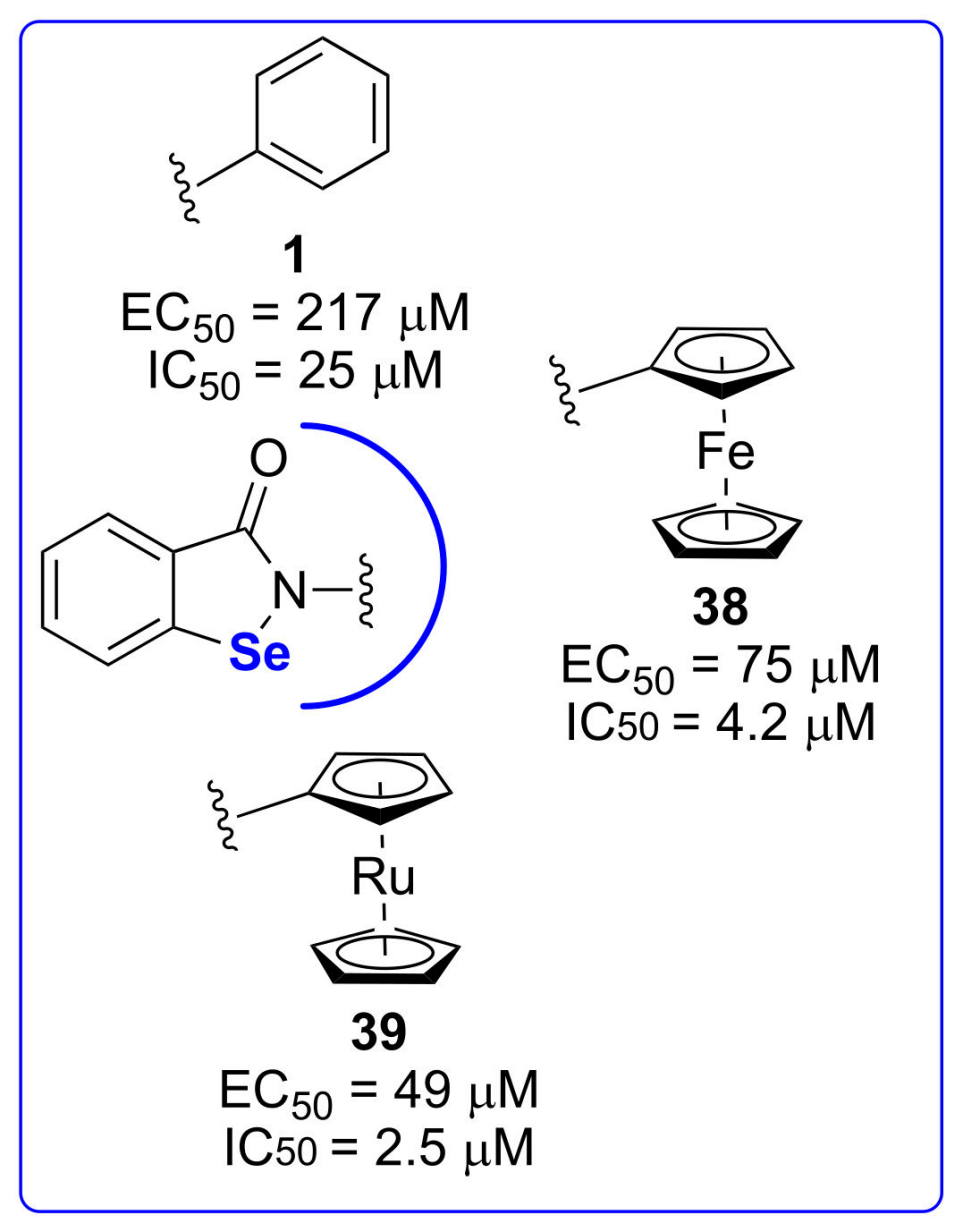
Structure of TR inhibitors. EC_50_ is the ability of compounds to inhibit MCF-7 growth while IC_50_ refers to the anti-TR activity.

Among the whole class of selenorganic compounds [112,113], the most promising derivative in the anticancer research field is ethaselen (compound **27**, Figure 3), which is under clinical evaluation in TR, overexpressing non-small cell lung cancers [114]. Ebselen and ebsulfur are also proven to interfere with TR [115] and in 2018 their scaffolds were used as a template for the synthesis of novel TR inhibitors. In particular, the N-phenyl ring was replaced with a metallocenyl group, increasing the benzisoselenazolone antiproliferative activity tested in MCF-7 cells, a model of breast cancer (Figure 9). Interestingly, the anti-TR activity well correlates with the compound’s ability to inhibit MCF-7 growth [116].

Beside TR, ebselen is proven to interfere with other targets to exert anti-proliferative activity. As an example, when administered to rat pancreatic tumor AR42J cells, it led to the phosphorylation of crucial components in mitogen-activated protein kinase (MAPK) pathways and oxidative stress at the level of endoplasmic reticulum. The combination of such effects ultimately results in cancer cell death [117]. 

6-Phosphogluconate dehydrogenase (6PGD) is a key enzyme in the pentose phosphate pathway which is overexpressed in several cancers and linked with enhanced cell growth and drug resistance. Very recently, ebselen was found to inhibit, with nanomolar potency, the activity of 6PGD, even in the context of cellular models of cancer. The enzymatic inhibitory activity translated into the ability to inhibit the growth of 6PGD overexpressing cells. Interestingly, the activity of the selenorganic compound was also evident in a xenograft mouse model [118]. 

**Figure 10:**
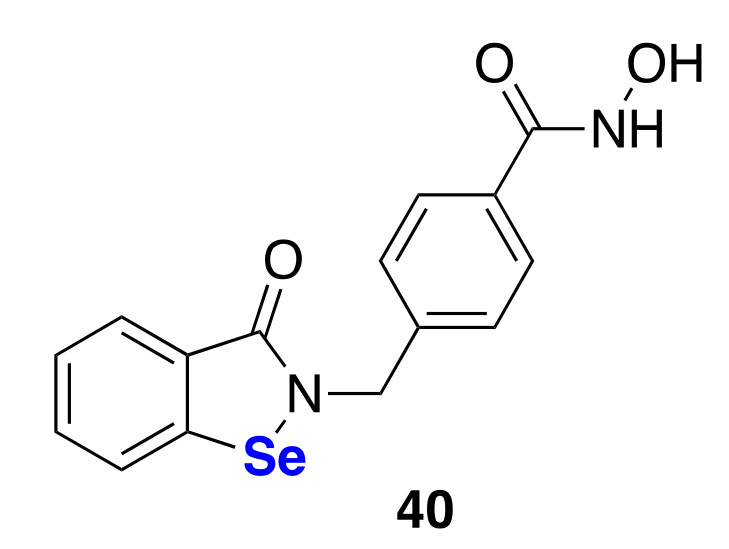
Structure of HDAC6 inhibitor.

Histone deacetylases (HDACs) are a class of enzymes very important for the synthesis of DNA since they remove acetyl groups from lysine residues of histones, improving the stability of the histones/DNA complex and negatively influencing DNA transcription [119]. Being a key regulator of gene expression, HDACs are a recognized target for the development of anticancer drugs. In humans, HDACs comprise a family of 18 members with different roles in the cells, so the real goal is the discovery of subtype selective HDAC inhibitors. With this in mind, Ma et al. recently reported that ebselen is a pan-HDAC inhibitor while compound **40** (Figure 10) is able to block HDAC6 with low nanomolar potency. Such enzymatic inhibitory activity translates into the ability to inhibit the growth of several models of cancer [120].

**Figure 11:**
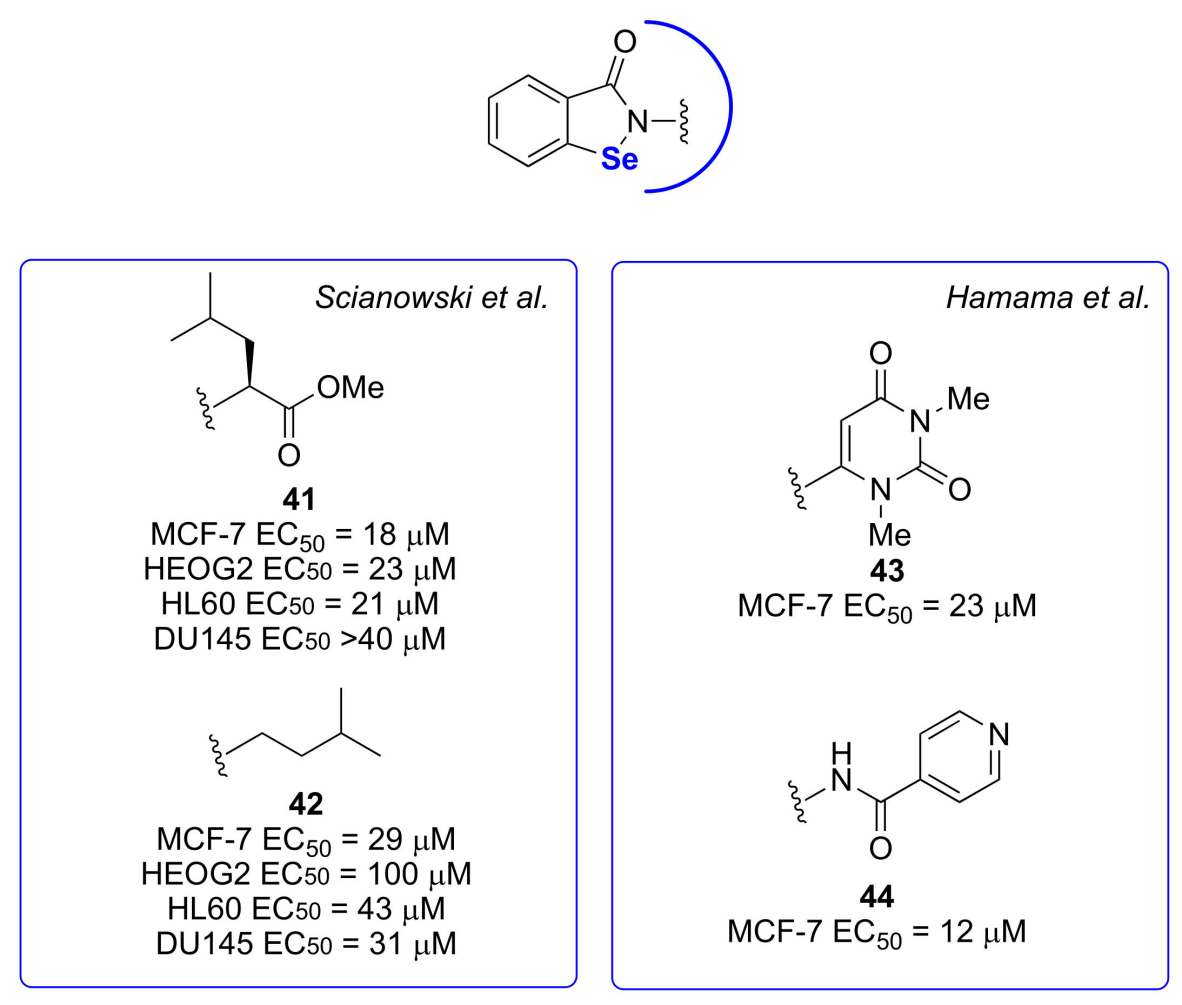
Compounds endowed with antiproliferative activities.

Among the medicinal chemistry projects focused on the manipulation of the benzisoselenazolone scaffold, in 2017 Scianowski et al. reported a switchable synthetic procedure to prepare both diselenides as well as ebselen-like structures, the latter were then screened against a panel of cancer cell lines. Worth mentioning are compounds **41** and **42** (Figure 11), which exerted a broad antiproliferative activity, being active toward models of breast carcinoma (MCF-7), liver cancer (HEPG2 cells), human promyelocytic leukemia (HL60 cells) and prostate cancer (DU145 cells). While compound **41** showed a fairly selective anti-proliferative activity, the methylbutyl-containing compound **42** was also able to exert cytotoxicity when tested in a non-cancerous PNT1A cell line [121,122]. The dynamic of the anti-prostate cancer inhibition of compound **42** was later studied in detail in a successive report by the same author [123]. 

Compounds **43** and **44** (Figure 11) were reported the same year by Hamama et al.; also in this case no selectivity was observed, the compounds being endowed with a low micromolar anti-proliferative activity measured in the MCF-7 cell line and in the normal human lung fibroblast cell line (WI-38) [124].

The MCF-7 cell line is a valuable model of breast cancer widely used to assess ebselen’s anti-proliferative properties. In this context, it was proven that 25 μg of compound **1** in combination with γ-radiation (6 Gy) has a significantly synergistic anti-proliferative effect. Such combination modulates gene expression, and inflammatory cytokines’ response, which results in the induction of apoptosis [125].

## 5. Anti-Neurodegenerative Disorders

### 5.1. Anti-Alzheimer’s Disease

Alzheimer’s disease (AD) is a devastating neurodegenerative disorder which is the leading cause of dementia in Western societies. Currently, the standard of care is purely palliative; indeed, the pharmacological treatments are unable to slow down the damage and destruction of neurons that cause Alzheimer’s symptoms and make the disease fatal. Even if the etiology of AD is still unknown, multiple factors are recognized to play a role in its pathogenesis; among these oxidative stress, inflammation, low levels of acetylcholine, τ-protein aggregation and β-amyloid (Aβ) deposits are recognized features [126,127].

In 2017, Liu et al. investigated the effects of a compound on AD-associated pathology and cognitive dysfunction using an AD model cell line, primary neurons and an AD mouse model. According to their results, ebselen was able to improve cognitive impairment, reduce the level of the most toxic soluble Aβ oligomers and inhibit τ hyperphosphorylation [128]. Later, compound **1** was tested against two acetylcholinesterase (AChE) isoforms and, at the same time, in a mouse model of amnesia by Nogueira et al. It was found that ebselen is a potent blocker of the AChE/G4 in vitro and elicits an antiamnesic effect in a scopolamine mouse model at relatively low concentrations [129]. The same research team tested ebselen in a streptozocin-induced mouse model of sporadic AD. The compound was administered intraperitoneally at the dosage of 10 mg/kg, proving several positive effects such as the reversal of memory impairment, antioxidant and anti-apoptotic effects that were superimposable to those elicited by the clinically used drug donepezil [130,131]. 

**Figure 12:**
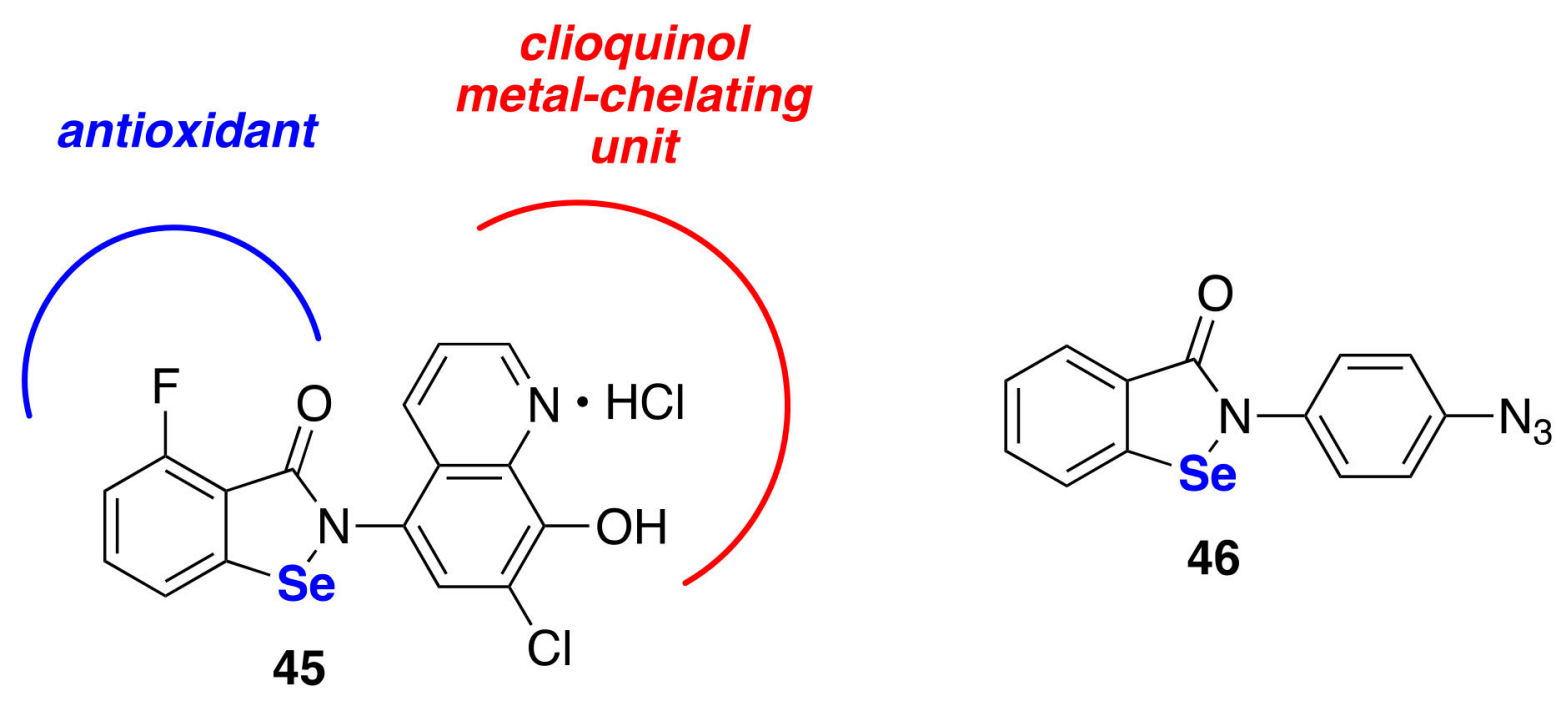
Structure of anti-AD compounds.

Since 2013, in light of its antioxidant activity, ebselen was used as a molecular platform for the development of anti-AD agents. It was merged with donepenzil in order to obtain multi-target-directed ligands (MTDL) capable of inhibiting both cholinesterases and oxidative stress simultaneously [132]. The synthesis of MTDL, also known as designed multiple ligands [133], is a new avenue in medicinal chemistry used not only in AD but also in several other research fields [134,135,136]. Recently, the benzisoselenazolone scaffold was merged with clioquinol, a compound known for its ability to chelate metals and inhibit Aβ deposits. Among the synthesized compounds, derivative **45** (Figure 12) exhibited the most potent inhibition of Aβ aggregation and excellent antioxidant activity. The compound was able to penetrate the CNS without showing toxicity in vivo [137]. 

Very recently, Sucheck et al. prepared a series of benzisoselenazolone derivatives identifying compounds **20** (Figure 2) and **46** (Figure 12) as promising neuroprotective compounds being able to protect human neuroblastoma SH-SY5Y cells against insult induced by an oxygen−glucose deprivation/reperfusion assay as a model of ischemia. These compounds behaved better than ebselen, taken as reference. Unfortunately, in vivo studies are lacking to definitely assess their actual clinical exploitability [138].

### 5.2. Anti-Amyotrophic Lateral Sclerosis

Amyotrophic lateral sclerosis (ALS) is a neurodegenerative disorder that affects the nervous system, leading to the degeneration of the spinal cord, that results in muscle weakness and atrophy, and respiratory failure. Unfortunately, with no effective cure available, ALS is fatal [139]. Evidence has clearly demonstrated that mutations to the gene encoding for superoxide dismutase-1 (SOD1) are associated with a subset of ALS [140]. The A4V substitution is one of the most severe mutations, causing destabilization and aggregation in ALS patients.

**Figure 13:**
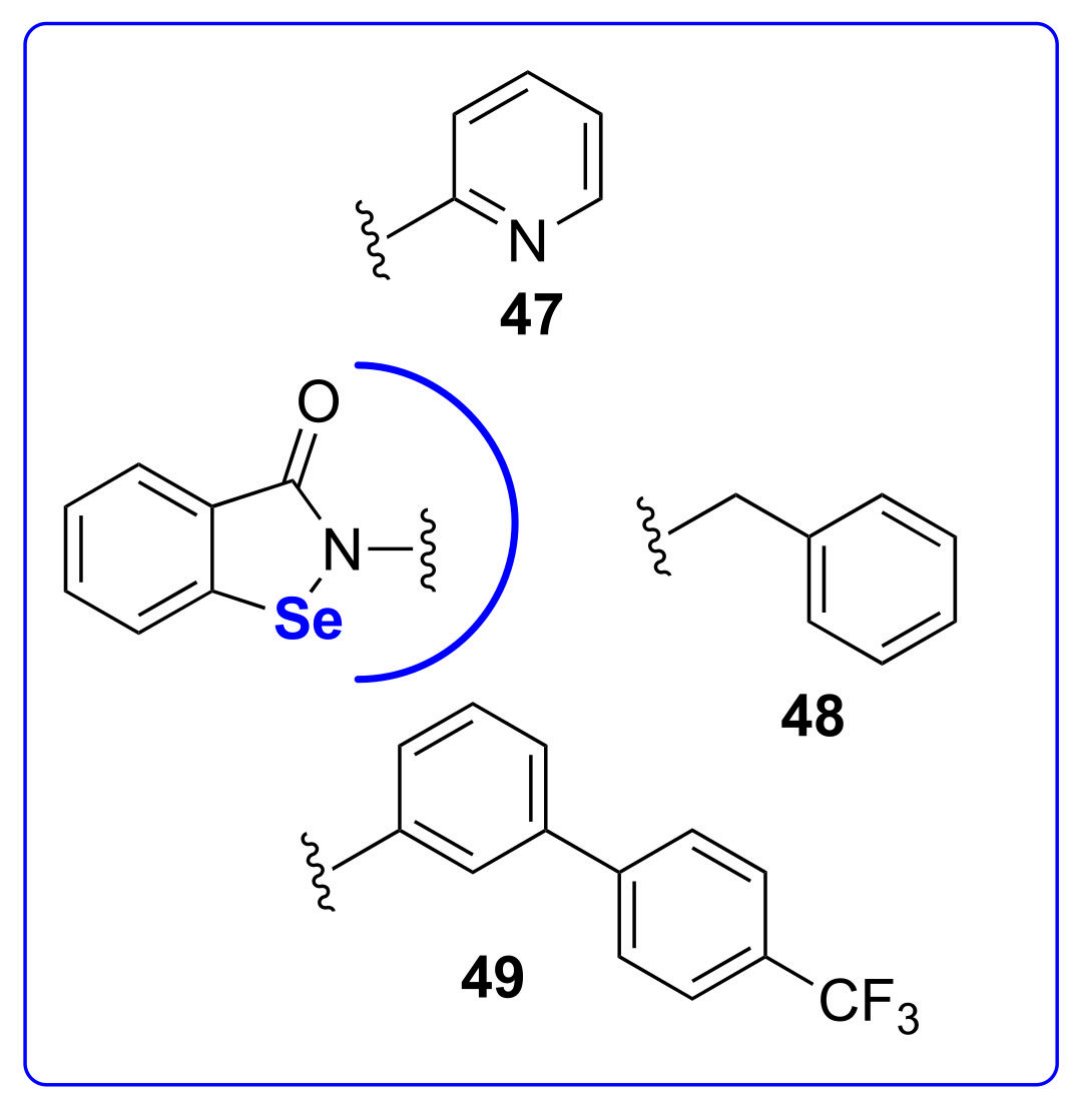
SOD1 Cys111 modifiers.

In 2018, Hasnain et al., through a drug repurposing approach, meant to discover “orphan diseases” treatments, found ebselen and ebsulfur (compound **23**) as SOD1 binders. They both link to Cys111, promoting the correct SOD1 folding and zinc binding and preventing its premature degradation and aggregation, ultimately hampering its toxicity. In light of this, ebselen can be considered as a valuable alternative to the recently approved drug edavarone [141]. Later, the same research group engaged in a broader study where they prepared a series of benzisoselenazolone and thiazolone analogues, identifying compounds **47–49** (Figure 13) as potent Cys111 modifiers. For each compound, suitable crystal structures were obtained, and this is really important from a de novo drug design perspective [142,143]. 

## 6. Anti-Bipolar Disorders

Bipolar disorder surely represents a serious health threat worldwide, particularly in the developed countries. Lithium carbonate is the only treatment which is able to act as mood stabilizer, controlling both mania and depression while decreasing suicide rates [144]. Unfortunately, the lithium mechanism of action is still unknown even if the so-called Berridge’s ”inositol depletion” hypothesis delivers a valid mechanistic explanation. It relies on the metal’s ability to normalize the signaling in overactive neurons by inhibiting inositol monophosphatase (IMPase) and depleting *myo*-inositol [145]. With this in mind, the Churchill group proved that ebselen inhibited IMPase and caused a lithium-like effect in mice [146] then, they proved that the compound was able to exert the same effect in humans. In particular, ebselen was tested in a randomized, double-blind, placebo-controlled, crossover design administered orally (3 × 200 mg capsules). As assessed by proton nuclear magnetic resonance, it slightly reduced *myo*-inositol levels in the anterior cingulate cortex, an anatomical region associated with emotional processing and bipolar disorder; in addition, it affected emotional processing in vivo [147]. In a successive investigation, they confirmed the above findings, finding also that the selenorganic compound was able to lower the glutaminase activity, further demonstrating ebselen’s viability as a treatment for bipolar disorder [148,149]. When challenged with the Cambridge Gambling Task, people receiving ebselen (3600 mg over 24 h) proved to have decreased impulsivity [150], confirming what was initially observed in a rodent model [151]. In different rodent models, compound **1** proved to reduce pilocarpine-mediated neural activation where lithium did the opposite. The ability to lower the muscarinic tone could explain the better tolerability of the selenorganic compound with respect to the metal when administered clinically [152].

The direct interaction between ebselen and IMPase was demonstrated in 2020 by Fenn et al., who solved the 1.47 Å resolution crystal structure of compound **1**/IMPase complex, in which a covalent bond links the electrophilic selenium to Cys141 [153].

Another randomized, double-blind, placebo-controlled clinical trial took place between October 2017 and June 2019, where ebselen was administered for 3 weeks at the dose of 600 mg twice a day to patients experiencing mania or hypomania. It proved to be numerically, but not statistically, superior to placebo, especially in those people who concomitantly received valproate treatment [154].

Like lithium carbonate, ebselen decreased 5-HT_2A_ receptor function in in vivo behavioral models and in vitro molecular models, further evidencing its potential role as an antidepressant in the treatment of bipolar disorder [155].

## 7. Anti-Hearing Loss/Ototoxicity

The hearing loss induced by noise is a major determinant of injury and disease in many teenagers and young adults. The reason behind this is the use of personal music players at relatively high volume and exposure to loud sounds, in particular in environments such as bars and clubs [156]. Evidence clearly indicates that GPx1 has a role in maintaining normal cochlear functions in mammals; indeed, GPx1 depletion increases vulnerability to noise-induced ear damage [157]. Because of its ability to mimic the activity GPx, ebselen proved to reduce hearing damage in animal models [158,159,160] even if it was unable to prevent the cochlear damage triggered by 2-hydroxypropyl-β-cyclodextrin, which however exerts damage by an oxidative stress-independent fashion [161]. 

Between January 2013 and March 2014, ebselen was assayed in a single-center, randomized, double-blind, placebo-controlled phase 2 trial on 85 healthy adults meant to check its otoprotection capabilities in reducing the temporary threshold shifts (TTS). The outcome of this trial proved that the compound is effective in blocking the TTS at the dose of 400 mg [162]. 

Among insults leading to hearing loss, aminoglycosides are worth mentioning because of their central role in the antimicrobial treatment in the context of cystic fibrosis (CF) management. Tobramicin, inhaled or injected, is a common drug in CF patients even if its use is associated with cochleotoxicity. Very recently, ebselen was tested in vitro in coadministration with the aminoglycoside, proving to reduce its damage. The in vitro results were confirmed in vivo, in a mouse model of auditory brainstem responses [163]. 

In 2019, the covalent inhibition of the insulin-degrading enzyme (IC_50_ = 42 nM) was suggested as an ancillary mechanism confirming ebselen’s anti-hearing-loss capacities [164]. The investigational efforts focused on ebselen in this particular research field were recently collected in a review article by Sound Pharmaceutical [165].

## 8. Miscellaneous Activities

### 8.1. In Vivo

The treatment of myocardial infarction usually relies on coronary reperfusion therapy, which however initiates a chain of events that result in additional cell injury called ischemia–reperfusion injury (IRI) [166]. In 2019, ebselen’s ability to attenuate myocardial IRI was assessed in vivo; in addition, it improved cardiac dysfunction, reducing apoptosis seemingly by increasing the expression of Bcl-2 in myocardial cells [167].

In 2016, a study meant to determine ebselen’s ability to decrease oxidative stress and improve endothelium-dependent vasodilation in patients with diabetes was carried out. In particular, a single-site, randomized, double-blind, placebo-controlled, crossover trial of 4 weeks in adults with diabetes and increased oxidative stress proved that ebselen was inactive in reducing markers of oxidative stress and arteriolar vascular function [168].

### 8.2. In Vitro

Ebselen was proved to inhibit in vitro a series of enzymes, thus paving the way for different therapeutic applications. Among the most recent examples, glutamate dehydrogenase (GDH) is an important enzyme in mitochondrial glutamate metabolism, which was found to be a potential target for the treatment of brain cancer. In a series of papers, Ruan et al. discovered that compound **1** is able to inhibit *E. coli* as well as human GDH [169,170]. Noteworthily, while the bacterial enzyme is inhibited covalently, the human isoform is bound reversibly. The reason behind this contrasting behavior is that the human isoform has an alanine residue in the place of a cysteine, capable of establishing a selenylsulfide bond with the selenorganic compound [171]. 

**Figure 14:**
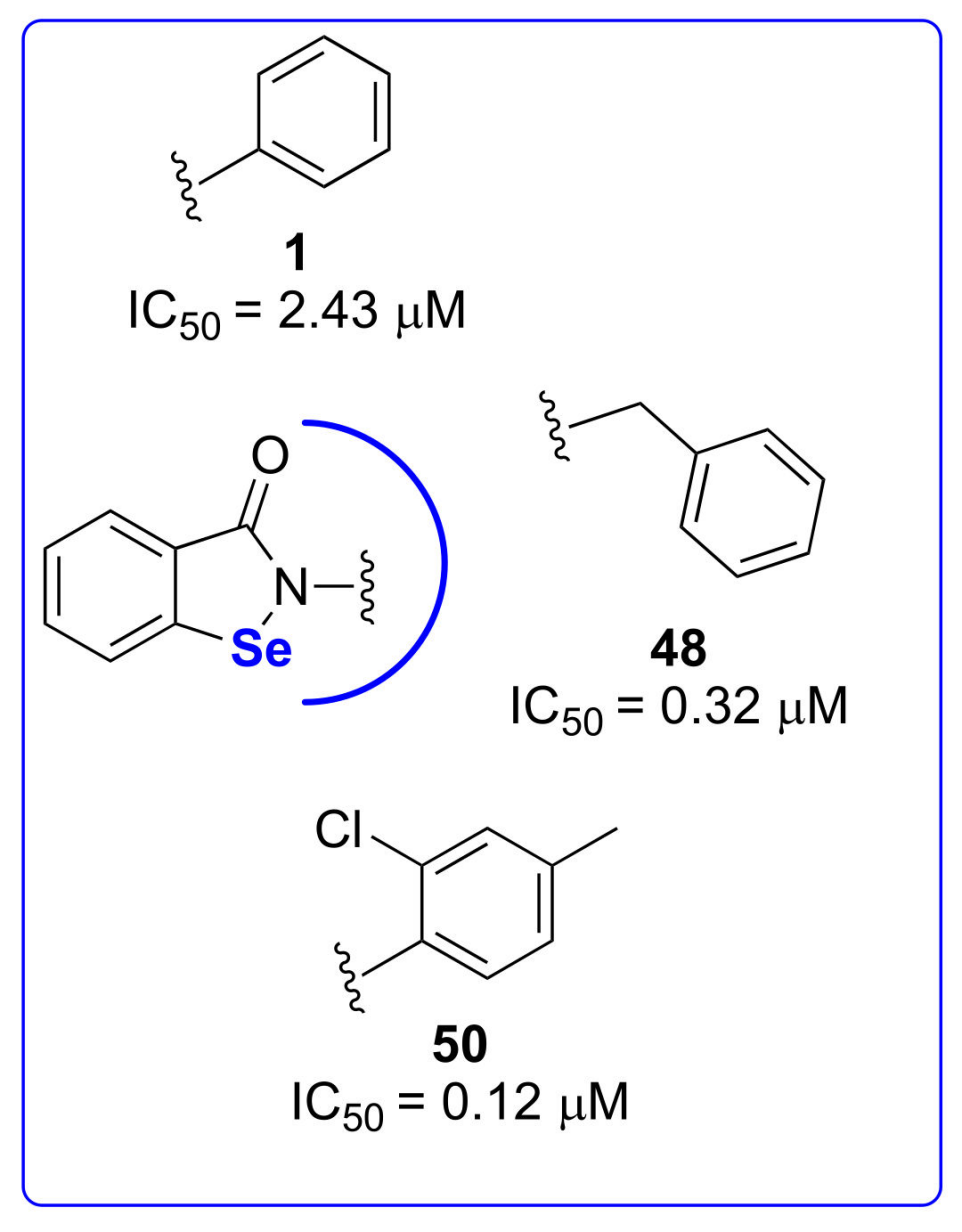
Structures and activity of MetAP2 inhibitors.

Methionine aminopeptidases (MetAPs) are neutral aminopeptidases containing metal ions that catalyze the removal of amino acids from the N-terminus of nascent peptides or proteins. Human MetAP type 2 (MetAP2) is one of the three known methionine aminopeptidases, linked to several types of cancer, which was the target of the drug discovery project by Weglarz-Tomczak et al. Starting with ebselen, a series of benzisoselenazolone and diselenide derivatives was prepared with compounds **48** and **50** bearing benzyl and 2-chloro-4-methyl phenyl fragments, respectively (Figure 14) resulting in the best-in-class being able to inhibit MetAP2 with a higher potency compared with ebselen [172].

## 9. Conclusions

Starting from its synthesis and successive biological evaluation, ebselen can be surely indicated as the most inspiring among the selenorganic compounds. It is currently taken as a reference in several pharmacological investigations even if it is now clear that it acts as what medicinal chemists name as a pan-assay interference compound (PAIN) because of its ability to bind non-selectively to reactive cysteines, being nucleophilically attached by the aminoacidic thiol functionality to the electrophilic selenium. As a proof of this, Tang and colleagues performed a sort of target fishing investigation by designing and synthesizing a biotinylated version of ebselen. When given to HeLa cell lysates, biotinylated ebselen was able to bind 462 proteins [173]. If from one side this peculiar chemical reactivity biases the perception of ebselen and its analogues in the medicinal chemistry community, one has to consider that this lack of specificity does not correspond with toxicity, ebselen being tested in several clinical trials without showing signs of acute or long-term toxicity. In the authors’ opinion, while ebselen is well suited as a pharmacological tool, the benzisoselenazolone scaffold offers a unique opportunity to design compounds able to potently, likely covalently, inhibit proteins containing cysteines in strategic positions without exerting toxic effects.
